# Short-term and long-term risk of mortality and neurodevelopmental impairments after bacterial meningitis during infancy in children in Denmark and the Netherlands: a nationwide matched cohort study

**DOI:** 10.1016/S2352-4642(22)00155-9

**Published:** 2022-09

**Authors:** Linde Snoek, Bronner P Gonçalves, Erzsébet Horváth-Puhó, Merel N van Kassel, Simon R Procter, Kirstine K Søgaard, Jaya Chandna, Arie van der Ende, Diederik van de Beek, Matthijs C Brouwer, Henrik T Sørensen, Joy E Lawn, Merijn W Bijlsma

**Affiliations:** a Department of Neurology, Amsterdam University Medical Centre, University of Amsterdam, Amsterdam, Netherlands; b Department of Medical Microbiology and Infection Prevention, Amsterdam Infection and Immunity, Amsterdam University Medical Centre, University of Amsterdam, Amsterdam, Netherlands; c Netherlands Reference Laboratory for Bacterial Meningitis, Amsterdam University Medical Centre, University of Amsterdam, Amsterdam, Netherlands; d Department of Paediatrics, Amsterdam University Medical Centre, University of Amsterdam, Amsterdam, Netherlands; f Amsterdam Neuroscience, Neuroinfection and Inflammation, Amsterdam, Netherlands; g Maternal, Adolescent, Reproductive and Child Health Centre and Department of Infectious Disease Epidemiology, London School of Hygiene & Tropical Medicine, London, UK; h Department of Clinical Epidemiology, Aarhus University Hospital and Aarhus University, Aarhus, Denmark; i Department of Clinical Microbiology, Aalborg University Hospital and Aalborg University, Aalborg, Denmark

## Abstract

**Background:**

Few studies have reported the long-term consequences of bacterial meningitis during infancy, and studies that have been done usually do not include a comparison cohort. We aimed to assess short-term and long-term risk of mortality, neurodevelopmental impairment (NDI), and health-care use and household income in cohorts of children with and without a history of bacterial meningitis during infancy in Denmark and the Netherlands.

**Methods:**

In this nationwide cohort study, infants with a history of bacterial meningitis before age 1 year were identified through the Danish Medical Birth Registry and Danish National Patient Registry using International Classification of Diseases (ICD)-10 codes and through the Netherlands Reference Laboratory for Bacterial Meningitis. Infants were matched (1:10) by sex and birth month and year to a comparison cohort of the general population without a history of bacterial meningitis. We analysed mortality using Cox proportional hazards regression. In Denmark, diagnoses of NDIs were based on ICD-10 codes; in the Netherlands, special educational needs were used as a functional NDI outcome. Risk ratios (RRs) of NDIs were estimated using modified Poisson regression. We also analysed long-term health-care use in Denmark and household income in both countries. All regression analyses were adjusted for sex and year of birth, and stratified by pathogen whenever sample size allowed.

**Findings:**

We included 2216 children with a history of bacterial meningitis (570 [25·7%] in Denmark between Jan 1, 1997, and Dec 31, 2018, and 1646 [74·3%] in the Netherlands between Jan 1, 1995, and Dec 31, 2018), matched to 22 127 comparison cohort members. Median age at diagnosis was 2·8 months (IQR 0·4–7·1) in Denmark and 4·3 months (0·7–7·4) in the Netherlands. Mortality risks within 3 months after disease onset were 3·9% (95% CI 2·6–5·8%) in Denmark and 5·9% (4·7–7·0) in the Netherlands, compared with 0·0% (p<0·0001) and 0·1% (p<0·0001) in the comparison cohorts. Survivors had an increased risk of moderate or severe NDIs at age 10 years (RR 5·0 [95% CI 3·5–7·1] in Denmark and 4·9 [4·0–6·2] in the Netherlands) compared to children in the comparison cohort, particularly after pneumococcal and group B streptococcal meningitis. In Denmark, a history of bacterial meningitis was associated with increased health-care use in the 10 years following diagnosis (rate ratio 4·5 [95% CI 3·9–5·2] for outpatient visits and 4·1 [3·6–4·7] for hospital admissions).

**Interpretation:**

Our study shows increased risk of mortality in the short and long term, a five times increase in risk of NDIs, and increased health-care use after bacterial meningitis during infancy. Together with context-specific incidence data, our results can advance pathogen-specific estimation of the meningitis burden and inform service provision at the individual and population level.

**Funding:**

Bill & Melinda Gates Foundation, the Stichting Remmert Adriaan Laan Fonds, and the Netherlands Organisation for Health Research and Development.

## Introduction

Bacterial meningitis is a life-threatening infection of the meninges and the subarachnoid space that causes substantial mortality and morbidity.^1^ Routine use of protein polysaccharide conjugates against *Haemophilus influenzae* type B, several serotypes of *Streptococcus pneumoniae,* and specific serogroups of *Neisseria meningitidis* in paediatric immunisation programmes has led to a decrease in the incidence of bacterial meningitis.^2^, ^3^ However, with an estimated 548 cases and 48 deaths from meningitis per 100 000 infants worldwide in 2019, the condition remains a serious threat to global health.^4^

In addition, bacterial meningitis during infancy has been associated with a high risk of sequelae in later life. Thus, it is a public health priority to characterise the long-term health burden of meningitis after its acute phase.^5^, ^6^, ^7^ Previous studies have suggested that individuals who have survived meningitis are at increased risk of dying after hospital discharge, either as a direct consequence of the acute episode or as a result of complications linked to the development of severe neurodevelopmental impairments (NDIs).^8^, ^9^, ^10^ Pathogen-specific estimates of health risks following bacterial meningitis are essential for patient counselling, health service planning, and evaluating the effects and cost-effectiveness of new interventions. Furthermore, as immunisation programmes have changed the distribution of causative pathogens, studies are necessary to characterise the current epidemiology of bacterial meningitis. However, studies on the outcomes of childhood bacterial meningitis into adolescence are rare, and usually do not include a comparison cohort.^6^, ^11^, ^12^ Cohort studies over several decades are challenging to undertake, but linkage of large databases can enable long follow-up and high matching ratios. Our multicountry collaboration used electronic cohorts to study long-term follow-up after invasive disease with a single pathogen, *Streptococcus agalactiae* (also known as group B *Streptococcus*);^13^ however, few follow-up studies have analysed multiple pathogens. We aimed to address these data gaps by assessing overall and pathogen-specific mortality in the short and long term, estimating risk of NDIs up to adolescence, and investigating long-term health-care use and household income in cohorts of children with and without a history of bacterial meningitis during infancy in Denmark and the Netherlands.

**Research in context**

**Evidence before this study**

Despite availability of vaccines that protect against several major pathogens, bacterial meningitis remains an important cause of mortality worldwide. Although acute mortality from this condition has been described in many settings, few studies have reported its long-term effects on health. A meta-analysis published in 2010 showed that the risk of neurodevelopmental impairments (NDIs) in both children and adults after bacterial meningitis was high, but varied by region and was pathogen-specific. In 2011, a systematic review on the long-term sequelae of bacterial meningitis in children aged 1 month to 18 years identified a few studies, with data on a total of 1433 survivors followed up for longer than 5 years. However, many of the studies included in the review were performed more than 10 years ago, included few children, had short follow-up, and did not include a comparison group. We searched PubMed for studies on long-term outcomes after bacterial meningitis during infancy, published in English from Jan 1, 2011, when the previous systematic review was published, to June 13, 2022, using the search terms: (bacterial meningitis) AND (infancy OR infant) AND (neurodevelopmental OR long-term). We found no other relevant papers that reported pathogen-specific, long-term follow-up data after bacterial meningitis during infancy and that recruited a comparison group.

**Added value of this study**

In this nationwide cohort study in two high-income countries, we identified 2216 infants diagnosed with bacterial meningitis in their first year of life (570 in Denmark and 1646 in the Netherlands) and matched them to 22 127 infants without a history of bacterial meningitis. Data on causative pathogens were available from International Classification of Diseases-10 codes in Denmark and results from microbiology cultures were available in the Netherlands. In both countries, risk of mortality within the first 3 months after bacterial meningitis diagnosis was considerably higher than the risk observed in the comparison cohorts. Although the absolute number of deaths was low after the acute period, children with a history of bacterial meningitis remained at increased risk of mortality after surviving the first month. Furthermore, NDIs were a frequent outcome for children with a history of bacterial meningitis, especially after *Streptococcus agalactiae* or pneumococcal meningitis infections. The percentage of children diagnosed with a NDI increased with age: over 10% of patients followed up for at least 10 years had moderate or severe NDIs. Consistent with these long-term health consequences, two-thirds of children with a history of bacterial meningitis were seen in outpatient clinic visits 1–5 years after diagnosis, compared with a third of children in the comparison cohort.

**Implications of all the available evidence**

This study addresses an important data gap on the short-term and long-term effects of bacterial meningitis during infancy. Using nationwide data from countries with similar methods but difference exposure and NDI outcomes, we provide robust evidence for the severe consequences of bacterial meningitis on the lives of affected children, which can become apparent from infancy up to adolescence. These data highlight the need for continued clinical care after hospitalisation for children with bacterial meningitis and for trials to compare therapeutic approaches that can reduce the risk of sequelae. Our findings provide the basis for future quantification of the relative burden caused by different bacterial pathogens of meningitis.

## Methods

### Study design and participants

We did a nationwide cohort study of infants with a history of bacterial meningitis, with disease onset before age 1 year, in Denmark and the Netherlands.

In Denmark, which has a free tax-supported health-care system,^14^ infants with a history of bacterial meningitis were identified through the Danish Medical Birth Registry and Danish National Patient Registry covering all Danish hospitals, on the basis of discharge diagnoses between Jan 1, 1997, and Dec 31, 2018, using International Classification of Diseases (ICD)-10 codes (appendix p 8).^14^, ^15^ In the Netherlands, infants with a history of culture-positive bacterial meningitis between Jan 1, 1995, and Dec 31, 2018, were identified through the Netherlands Reference Laboratory for Bacterial Meningitis (NRLBM),^16^ which receives approximately 90% of isolates cultured from blood and cerebrospinal fluid (CSF).^13^, ^17^ The NRLBM dataset was linked to the PeriNed perinatal registry^18^ and the Dutch Hospital Data Registry. More information on these national databases is provided in the appendix (pp 5–6).

Members of the comparison cohorts were randomly selected without replacement and matched 10:1 on sex and year and month of birth to each child in the cohort with a history of bacterial meningitis. To be eligible for matching, children in the comparison cohort had to be alive and to have no diagnosis of bacterial meningitis before their index date. In Denmark, children in the comparison cohort were selected using the Danish Medical Birth Registry^19^ and the Danish Civil Registration System.^20^ In the Netherlands, the Municipal Personal Records Database^21^ was used.

This study was reported to the Danish Data Protection Agency (record number 2015–57–0002). In the Netherlands, our research (EPI-408) was submitted to the Centre for Clinical Expertise at the National Institute for Public Health and the Environment and was exempted from further approval by an ethical research committee, according to Dutch law for medical research involving humans. Furthermore, we had permission to use the data provided by Statistics Netherlands for our study purposes.

### Procedures and outcomes

We present separate analyses for each country because of differences in case definitions. All infants were followed up from date of diagnosis of bacterial meningitis until death, emigration, or end of study period (Dec 31, 2018, in Denmark and Dec 31, 2019, in the Netherlands), whichever came first. Data on sex, year of birth, gestational age, birthweight, and maternal age were obtained from the Danish Medical Birth Registry and Danish Civil Registration System for the cohorts in Denmark and the Netherlands Reference Laboratory for Bacterial Meningitis, PeriNed Perinatal Registry, and Dutch Municipal Personal Records Registry for the cohorts in the Netherlands. In the Netherlands, data on gestational age and birthweight were only available for children born after Dec 31, 1999. In Denmark, the first hospital admission date for bacterial meningitis was used to calculate age at disease onset. In the Netherlands, the first date of a positive culture was used to calculate this age. If this date was not reported in the Netherlands, we used the date that the isolate was submitted to or received by the NRLBM.

First, we assessed overall and pathogen-specific all-cause mortality in the short term (3 months) and long term (5 years and 10 years) in the cohorts with a history of bacterial meningitis, compared with mortality in the comparison cohorts, using data from the Danish Civil Registration System^20^ and the Dutch Municipal Personal Records Database.^21^

Second, we compared the risk of NDIs in adolescents aged up to 20 years in Denmark and in children aged up to 11 years in the Netherlands. We describe 5-year and 10-year estimates given that these estimates were available for both countries. Different definitions of NDI were used in the two countries. In Denmark, a diagnosis of NDI was based on ICD-10 codes for mental, behavioural, and nervous system disorders with data from the Danish National Patient Registry.^15^ Motor, hearing, vision, cognitive, and social or behavioural domains were studied and impairments were categorised by severity (ie, mild, moderate, or severe) using ICD-10 codes (appendix pp 9–12). Any NDI was defined as an impairment in any of these domains and multi-domain NDIs were defined as impairments in more than one domain (appendix pp 9–12). In the Netherlands, data on special educational needs, which were obtained from the Dutch Primary School Registry and the Dutch Special Education School Registry (provided by Statistics Netherlands), were used as functional outcomes of NDI. Children who received additional support in regular schools were categorised as having mild NDI and children who received education in a special needs school were categorised as having a moderate or severe NDI (appendix pp 9–12). Risks of NDI and special educational needs were assessed at age 5 years and 10 years. To be included in the analyses, children had to be followed up for at least 5 years or 10 years, respectively.

Third, we quantified health-care use and household income in the cohorts of children with and without a history of bacterial meningitis. In Denmark, data on number of hospital admissions, length of hospital stays, and frequency of outpatient clinic visits (not including general practitioner or pharmacy visits) were obtained from the Danish National Patient Registry. These data were not available in the Netherlands. Household income was quantified in Denmark by summing the gross income of each child's parents from the Income Statistics Register and converting it into euros.^22^, ^23^ In the Netherlands, household income was derived from a registry database on standardised disposable household income after adjustment for family size and taxes. Incomes were inflated to 2019 currency values by use of the World Bank's gross domestic product deflator.^23^ In both countries, we assessed household income for each calendar year following the year of bacterial meningitis diagnosis.

### Statistical analysis

To calculate and compare risk of mortality, we used the Kaplan-Meier method and log-rank test. Hazard ratios were computed by Cox proportional hazards regression. To assess risk of post-acute mortality, 10-year mortality was estimated after excluding infants who died or were lost to follow-up within 30 days of the diagnosis of meningitis. To estimate risk ratios (RRs) of NDIs, we used modified Poisson regressions with robust variance estimators.^24^ To investigate the association between bacterial meningitis during infancy and health-care use in later life, we compared the number of hospital admissions and outpatient clinic visits between the patient cohort with a history of bacterial meningitis and the comparison cohort using negative-binomial regression models. Analyses of health-care use were restricted to children who survived at least 5 years and 10 years after the episode of bacterial meningitis. When assessing household income, we did not adjust for baseline income. For the cohort with a history of bacterial meningitis, descriptive income analyses were stratified by diagnoses of NDIs or death during follow-up.

All regression analyses were adjusted for sex and year of birth, and stratified by pathogen, whenever sample size allowed. Prematurity is a probable confounder of the association between meningitis and acute or long-term outcomes, particularly for pathogens that cause neonatal infections. To account for this potential confounding, we additionally adjusted for gestational age in a sensitivity analysis. In the Netherlands, analyses adjusted for gestational age were restricted to children born after 1999 because gestational age was not available for this study before 2000. In Denmark, children with missing gestational age were removed from these sensitivity analyses.

Statistical analyses were performed using SAS (version 9.4) in Denmark and SPSS (version 25.0) and Stata (version 16.0) in the Netherlands. Of note, the statistical approach used to study each of these outcomes closely parallels the previous analyses we reported on the long-term outcomes in children after invasive infection with group B *Streptococcus*, including sepsis.^13^

### Role of the funding source

The funder of the study had no role in study design, data collection, data analysis, data interpretation, or writing of the report.

## Results

**Figure 1:**
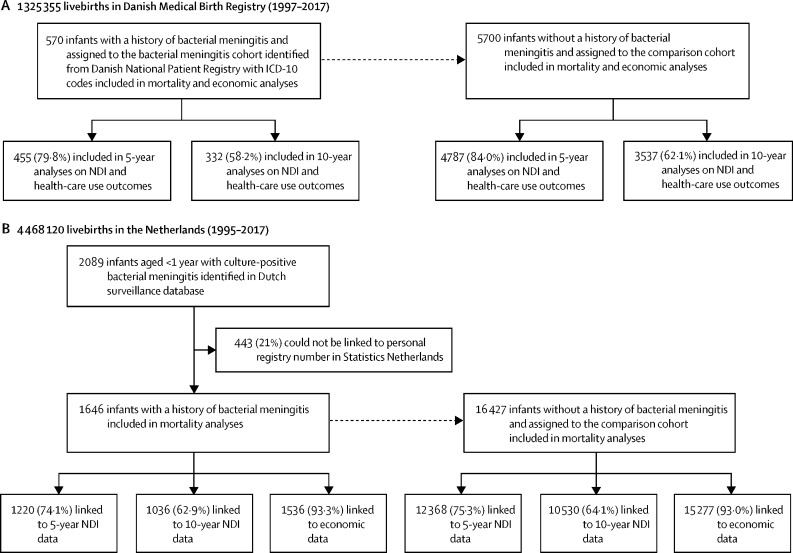
Participant selection Flowchart of the inclusion of children in the bacterial meningitis cohort and comparison cohort in Denmark (A) and the Netherlands (B). ICD=International Classification of Diseases. NDI=neurodevelopmental impairment.

**Figure 2:**
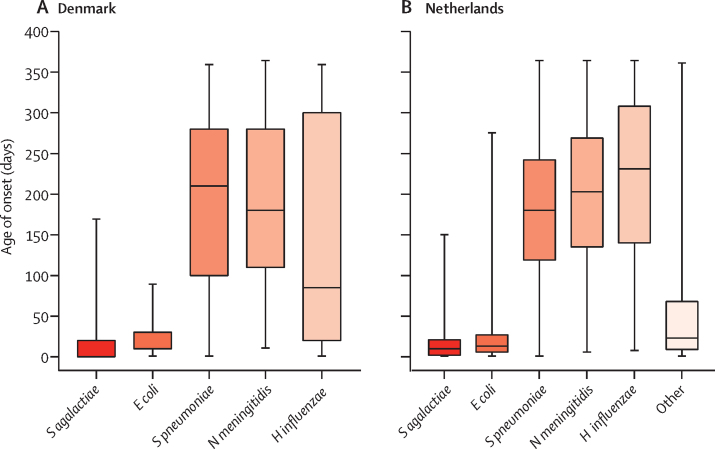
Median age of onset of meningitis Median age of onset in the cohort with a history of meningitis from Denmark (A) and the Netherlands (B), by causative agent. Causative agents include *Streptococcus agalactiae, Escherichia coli, Streptococcus pneumoniae, Neisseria meningitidis, Haemophilus influenzae*, and other (ie, less common, gram-positive, and gram-negative bacteria). Error bars represent minimum and maximum range.

Between Jan 1, 1997, and Dec 31, 2017, 1 325 355 livebirths were reported in Denmark. Between Jan 1, 1995, and Dec 31, 2017, 4 468 120 livebirths were reported in the Netherlands. Overall, we identified 2216 infants with a history of bacterial meningitis in the first year of life (570 [25·7%] in Denmark and 1646 [74·3%] in the Netherlands) and matched them to 22 127 infants without a history of bacterial meningitis (5700 in Denmark and 16 427 in the Netherlands; figure 1). The most common causative pathogens of bacterial meningitis were *S pneumoniae, S agalactiae*, and *N meningitidis* (table 1). Median age at diagnosis of bacterial meningitis was 2·8 months (IQR 0·4–7·1) in Denmark and 4·3 months (0·7–7·4) in the Netherlands. In the Netherlands, age of meningitis onset was based on the first culture date in 1433 (87·1%) cases and on the date that the isolate was submitted to or received by the NRLBM in 213 (12·9%) cases. The youngest median age of onset occurred in infants with *S agalactiae* meningitis (10 days [IQR 0–20] in Denmark and 10 days [2–21] in the Netherlands) or *Escherichia coli* meningitis (10 days [10–30] in Denmark and 13 days [6–27] in the Netherlands). In both countries, median age of onset was approximately 6 months in infants with pneumococcal (210 days [100–280] in Denmark and 180 days [119–242] in the Netherlands) or meningococcal meningitis (180 days [110–280] in Denmark and 203 days [135–269] in the Netherlands; figure 2).

**Table 1:** Baseline participant characteristics

		**Denmark (1997–2018)**		**The Netherlands (1995–2018)**	
		Bacterial meningitis cohort (n=570)	Comparison cohort (n=5700)	Bacterial meningitis cohort (n=1646)	Comparison cohort (n=16 427)
**Time period**					
1995–2000*		110 (19·3%)	1100 (19·3%)	608 (36·9%)	6062 (36·9%)
2001–2006		192 (33·7%)	1920 (33·7%)	552 (33·5%)	5511 (33·5%)
2007–2012		158 (27·7%)	1580 (27·7%)	264 (16·0%)	2638 (16·1%)
2013–2018		110 (19·3%)	1100 (19·3%)	222 (13·5%)	2216 (13·5%)
**Sex**					
	Female	255 (44·7%)	2550 (44·7%)	698 (42·4%)	6971 (42·4%)
	Male	315 (55·3%)	3150 (55·3%)	948 (57·6%)	9456 (57·6%)
**Gestational age, weeks**†					
	<28	11 (1·9%)	8 (0·1%)	24/1101 (2·2%)	21/10 992 (0·2%)
	28–37	114 (20·0%)	350 (6·1%)	152/1101 (13·8%)	696/10 992 (6·3%)
	≥37	431 (75·6%)	5216 (91·5%)	836/1101 (75·9%)	9141/10 992 (83·2%)
	Data missing	14 (2·5%)	126 (2·2%)	89/1101 (8·1%)	1134/10 992 (10·3%)
Birthweight, grams		3400 (2800–3800)	3516 (3162–3880)	3382 (2870–3770)	3456 (3090–3820)
	Data missing	10 (1·8%)	106 (1·9%)	81/1101 (7·4%)	1082/10 992 (9·8%)
Maternal age, years		30 (27–33)	30 (27–34)	30 (27–33)	31 (27–34)
	Data missing	0	0	0	0
**Causative pathogen of meningitis**					
*Streptococcus agalactiae*		177 (31·1%)	NA	325 (19·7%)	NA
*Streptococcus pneumoniae*		178 (31·2%)	NA	527 (32·0%)	NA
*Neisseria meningitidis*		142 (24·9%)	NA	477 (29·0%)	NA
*Escherichia coli*		58 (10·2%)	NA	145 (8·8%)	NA
*Haemophilus influenzae*		15 (2·6%)	NA	89 (5·4%)	NA
Other‡		NA	NA	83 (5·0%)	NA

Data are n (%), n/N (%), or median (IQR). NA=not applicable.

* The inclusion period was from Jan 1, 1995, to Dec 31, 2018, in the Netherlands and from Jan 1, 1997, to Dec 31, 2018, in Denmark.

† In the Netherlands, analyses adjusted for gestational age were restricted to children born after Dec 31, 1999, because gestational age was not available for this study before 2000.

‡ Other bacteria consisted of less common, gram-positive, and gram-negative bacteria. Due to Dutch data protection regulations, detailed information on these causative pathogens are not permitted.

In Denmark, 125 (21·9%) of 570 infants in the cohort with a history of bacterial meningitis were born preterm (<37 weeks), compared with 358 (6·3%) of 5700 in the comparison cohort (table 1). In the Netherlands, 176 (16·0%) of 1101 infants in the cohort with a history of bacterial meningitis and born after Dec 31, 1999, were born preterm, compared with 717 (6·5%) of 10 992 in the comparison cohort (table 1).

Overall, 20 (3·5%) children in Denmark and 86 (5·2%) children in the Netherlands died within 30 days of a diagnosis of bacterial meningitis. Infants with bacterial meningitis were followed up for a median of 12 years (IQR 6–17) in Denmark and 16 years (11–20) in the Netherlands. Risk of mortality during the first 3 months after disease onset in the cohorts with a history of bacterial meningitis was 3·9% (95% CI 2·6–5·8) in Denmark and 5·9% (4·7–7·0%) in the Netherlands (table 2). Risk of mortality during the first 3 months after disease onset in the comparison cohorts was lower: 0·0% (0·0–0·0; p<0·0001) in Denmark and 0·1% (0·1–0·1; p<0·0001) in the Netherlands. In Denmark, risk of mortality during the first 3 months after disease onset was high in infants with *E coli* meningitis (6·9% [2·7–17·3]) and in those with *H influenzae* meningitis (6·7% [1·0–38·7]). In the Netherlands, this risk was high in infants with *S agalactiae* meningitis (8·7% [5·8– 11·9]), followed by those with *E coli* meningitis (5·7% [2·8–10·7]). However, due to few or no deaths in the comparison cohorts matched to children with *S pneumoniae* or *N meningitidis* meningitis, the relative increase in mortality was high for these two pathogens (table 2). Associations between bacterial meningitis and mortality were also observed in analyses with longer follow-up (5 years and 10 years; table 2) and after adjustment for gestational age (appendix p 14). Risk of mortality within 10 years of disease onset in children who survived the first 30 days following onset of bacterial meningitis was 1·5% (95% CI 0·8–3·0) in Denmark and 3·4% (2·5–4·5) in the Netherlands, which was higher than in children in the comparison cohorts in both countries (0·1% [0·1–0·3] in Denmark and 0·3% [0·2–0·4] in the Netherlands; appendix p 15).

**Table 2:** Cumulative risk of mortality in children with and without a history of bacterial meningitis

		**Denmark**			**The Netherlands**			
	Bacterial meningitis cohort (95% CI)*	Comparison cohort (95% CI)	HR (95% CI)	p value	Bacterial meningitis cohort (95% CI)	Comparison cohort (95% CI)	HR (95% CI)	p value
**All pathogens**								
<3 months	3·9% (2·6–5·8)	0·0% (0·0–0·0)	..	..	5·9% (4·7–7·0)	0·1% (0·1–0·1)	74·1 (41·5–132·4)	<0·0001
<5 years	4·8% (3·3–6·9)	0·1% (0·0–0·2)	55·5 (21·4–144·1)	<0·0001	8·0% (6·5–9·1)	0·3% (0·2–0·4)	28·7 (20·1–41·0)	<0·0001
<10 years	4·8% (3·3–6·9)	0·1% (0·1–0·3)	46·3 (19·1–112·1)	<0·0001	10·1% (8·2–11·4)	0·3% (0·2–0·4)	24·1 (17·4–33·3)	<0·0001
**Streptococcus agalactiae**								
<3 months	3·9% (1·9–8·1)	0·0% (0·0–0·0)	..	..	8·7% (5·8–11·9)	0·3% (0·1–0·5)	35·2 (16·0–77·4)	<0·0001
<5 years	5·2% (2·7–9·7)	0·1% (0·01–0·4)	93·4 (11·8–736·9)	<0·0001	10·5% (7·0–14·1)	0·5% (0·3–0·8)	21·2 (11·0–41·0)	<0·0001
<10 years	5·2% (2·7–9·7)	0·1% (0·01–0·4)	93·4 (11·8–736·9)	<0·0001	17·6% (11·8–21·8)	0·7% (0·4–1·2)	21·0 (11·2–39·5)	<0·0001
**Streptococcus pneumoniae**								
<3 months	3·9% (1·9–8·1)	0·0% (0·0–0·0)	..	..	5·5% (3·7–7·6)	0·0% (0·0–0·0)	..	..
<5 years	4·5% (2·3–8·8)	0·1% (0·01–0·4)	81·7 (10·2–652·8)	<0·0001	8·5% (6·1–11·0)	0·2% (0·1–0·4)	45·6 (22·1–94·0)	<0·0001
<10 years	4·5% (2·3–8·8)	0·1% (0·03–0·5)	40·9 (8·7–192·7)	<0·0001	10·6% (7·6–13·3)	0·3% (0·1–0·5)	40·6 (21·0–78·7)	<0·0001
**Neisseria meningitidis**								
<3 months	2·1% (0·7–6·4)	0·0% (0·0–0·0)	..	..	4·9% (3·2–7·2)	0·0% (0·0–0·2)	116·6 (27·5–494·7)	<0·0001
<5 years	2·1% (0·7–6·4)	0·1% (0·04–0·6)	15·3 (2·6–91·6)	0·0028	5·6% (3·7–7·9)	0·2% (0·1–0·4)	28·0 (13·1–60·1)	<0·0001
<10 years	2·1% (0·7–6·4)	0·1% (0·04–0·6)	15·3 (2·6–91·6)	0·0028	6·2% (4·2–8·7)	0·4% (0·2–0·6)	17·5 (9·3–33·1)	<0·0001
**Haemophilus influenzae**								
<3 months	6·7% (1·0–38·7)	0·0% (0·0–0·0)	..	..	2·3% (0·6–8·7)	0·0% (0·0–0·0)	..	..
<5 years	13·9% (3·6–45·0)	0·0% (0·0–0·0)	..	..	3·7% (1·2–10·9)	0·0% (0·0–0·0)	..	..
<10 years	13·9% (3·6–45·0)	0·0% (0·0–0·0)	..	..	4·5% (1·4–12·9)	0·2% (0·02–1·1)	19·1 (1·8–206·6)	0·015
**Escherichia coli**								
<3 months	6·9% (2·7–17·3)	0·0% (0·0–0·0)	..	..	5·7% (2·8–10·7)	0·1% (0·0–0·6)	41·4 (8·8–194·8)	<0·0001
<5 years	8·7% (3·7–19·7)	0·2% (0·03–1·4)	52·2 (6·1–447·2)	0·0003	9·2% (5·0–15·3)	0·6% (0·3–1·2)	16·2 (6·3–41·8)	<0·0001
<10 years	8·7% (3·7–19·7)	0·2% (0·03–1·4)	52·2 (6·1–447·2)	0·0003	10·6% (7·6–13·3)	0·7% (0·3–1·5)	15·0 (5·8–38·8)	<0·0001

HRs are adjusted for matching variables (ie, sex and year of birth). HR=hazard ratio. In Denmark, the median duration of follow-up before death was 5 days (IQR 2–73) in the cohort with a history of bacterial meningitis and 3 years (1–12) in the comparison cohort. In the Netherlands, median duration of follow-up before death was 6 days (1–458) in the cohort with a history of bacterial meningitis and 4 years (0–16) in the comparison cohort. For children who survived the entire study period, median duration of follow-up for both cohorts was 12 years (7–17) in Denmark and 16 years (11–20) in the Netherlands.

* The mortality risk is the same for 5 years and 10 years in Denmark because none of the children in the cohort died in the 5–10 year period.

**Figure 3:**
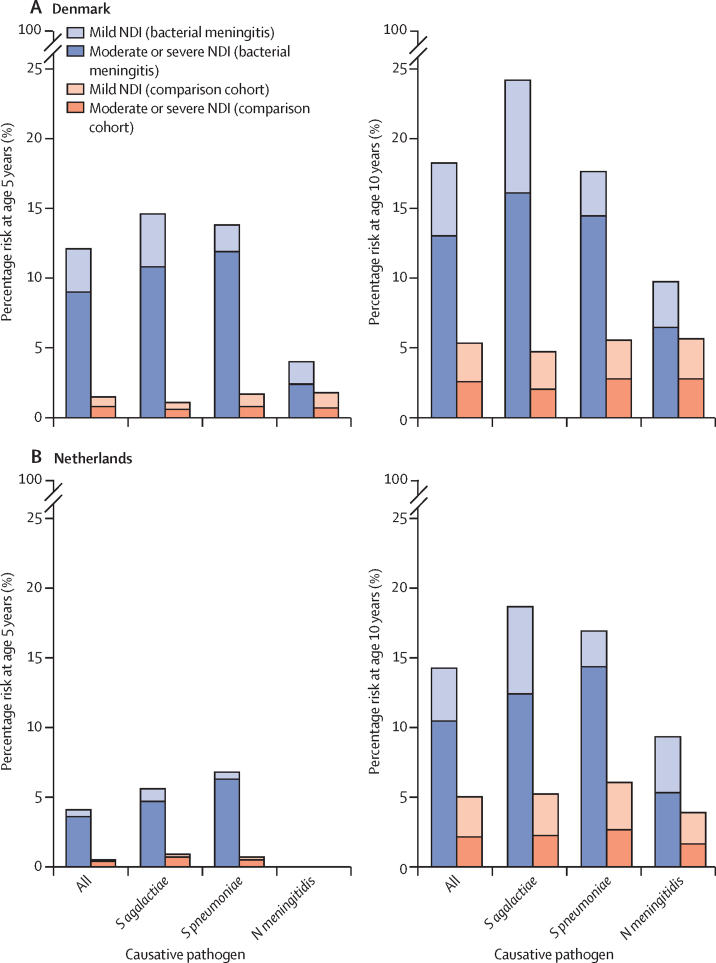
Risk of NDIs in children with and without a history of bacterial meningitis in Denmark and the Netherlands Risk of NDIs in Denmark (A) and in the Netherlands (B), by causative agent. Causative agents include *Streptococcus agalactiae, Streptococcus pneumoniae*, and *Neisseria meningitidis*. Due to low event numbers in relation to data protection regulations, data on NDIs in children with *N meningitidis* meningitis at age 5 years in the Netherlands and in children with *Haemophilus influenzae* and *Escherichia coli* meningitis at age 5 years and 10 years in both countries could not be reported. NDI=neurodevelopmental impairment.

**Figure 4:**
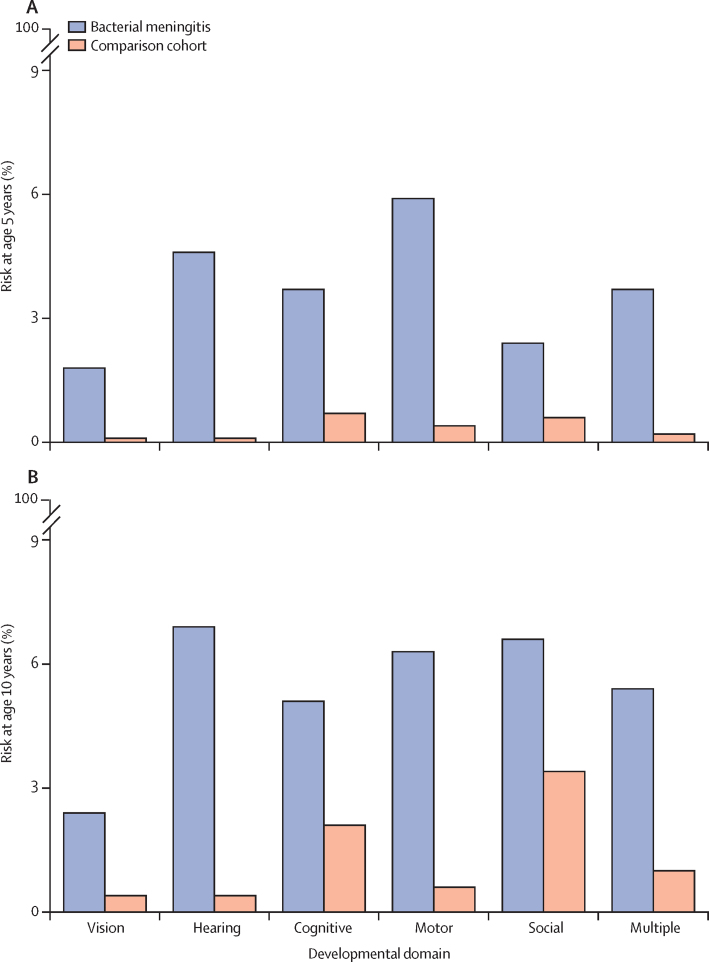
Domain-specific risk of NDIs in children with and without a history of meningitis in Denmark Risk of NDIs at age 5 years (A) and 10 years (B), by domain. NDI=neurodevelopmental impairment.

For the analyses of NDIs, 455 (79·8%) children in Denmark and 1220 (74·1%) children in the Netherlands were included in the 5-year analyses and 332 (58·2%) children in Denmark and 1036 (62·9%) children in the Netherlands were included in the 10-year analyses (figure 1). In Denmark, 42 (12·7%) of 332 children in the cohort with a history of bacterial meningitis had moderate or severe NDIs by age 10 years, compared with 89 (2·5%) of 3537 children in the comparison cohort (RR 5·0 [95% CI 3·5–7·1]; figure 3; table 3). In the Netherlands, 106 (10·2%) of 1036 children in the cohort with a history of bacterial meningitis had received education in a special needs school by age 10 years, compared with 217 (2·1%) of 10 530 children in the comparison cohort (4·9 [4·0–6·2]; table 3). After adjusting for gestational age, the RR of moderate or severe NDIs by age 10 years after bacterial meningitis was 4·9 (3·2–7·4) in Denmark and 3·9 (3·0–5·1) in the Netherlands (appendix p 16). In both countries, there were strong associations between moderate or severe NDIs by age 10 years and *S pneumoniae* meningitis (5·1 [3·0–8·8] in Denmark and 5·3 [3·9–7·4] in the Netherlands) and *S agalactiae* meningitis (7·9 [4·1–15·2] in Denmark and 5·4 [3·2–9·0] in the Netherlands; table 3). The most frequently affected domains in children with NDIs were hearing (23 [6·9%]) and social or behavioural (22 [6·6%]). Impairment in more than one domain occurred in 18 (5·4%) individuals with a history of bacterial meningitis (figure 4; appendix p 17). Infants with a history of meningitis caused by *S pneumoniae* or *S agalactiae* more often had impairments in the motor (5·5% and 11·2%) and hearing (12·5% and 4·5%) domains than did children with a history of meningitis caused by *N meningitidis* (1·1% motor and 2·1% hearing).

**Table 3:** RRs for NDI outcomes in children with and without a history of bacterial meningitis

	**All pathogens**		**Streptococcus agalactiae**		**Streptococcus pneumoniae**		**Neisseria meningitidis**	
	Any NDI	Moderate or severe NDI	Any NDI	Moderate or severe NDI	Any NDI	Moderate or severe NDI	Any NDI	Moderate or severe NDI
**Denmark**								
<5 years	7·8 (5·6–11·0)	11·7 (7·6–18·0)	12·8 (6·8–24·3)	18·9 (8·1–44·1)	8·2 (4·8–13·9)	14·1 (7·2–27·5)	2·2 (0·8–5·5)	3·4 (0·9–12·6)
<10 years	3·4 (2·6–4·5)	5·0 (3·5–7·1)	5·1 (3·2–8·3)	7·9 (4·1–15·2)	3·2 (2·0–4·9)	5·1 (3·0–8·8)	1·7 (0·9–3·4)	2·4 (1·0–5·7)
<15 years	2·5 (1·8–3·4)	3·5 (2·5–5·1)	3·5 (2·1–6·1)	5·4 (2·8–10·6)	2·1 (1·3–3·5)	3·0 (1·7–5·3)	1·9 (0·9–3·7)	2·2 (0·9–5·0)
<20 years	2·8 (1·6–4·9)	3·1 (1·5–6·5)	2·2 (0·5–8·9)	3·7 (0·8–16·5)	2·6 (1·1–6·5)	2·5 (0·8–8·2)	3·6 (1·4–9·1)	3·8 (1·1–13·3)
**The Netherlands**								
<5 years	7·5 (5·2–10·7)	8·8 (5·9–13·1)	6·2 (3·1–12·1)	6·4 (3·1–13·5)	10·2 (6·1–16·9)	13·4 (7·5–23·8)	3·7 (1·0–13·9)	3·3 (0·7–16·3)
<10 years	2·9 (2·4–3·4)	4·9 (4·0–6·2)	3·5 (2·4–5·2)	5·4 (3·2–9·0)	2·8 (2·1–3·6)	5·3 (3·9–7·4)	2·4 (1·7–3·5)	3·2 (1·9–5·5)
<11 years	2·5 (2·1–2·9)	4·6 (3·8–5·7)	3·1 (2·2–4·5)	5·7 (3·4–9·5)	2·6 (2·1–3·4)	5·3 (3·9–7·2)	2·1 (1·5–2·9)	3·0 (1·8–4·9)

Data are RR (95% CI). RRs for the association between history of bacterial meningitis and NDI outcomes were estimated with a modified Poisson regression model. RR=risk ratio. NDI=neurodevelopmental impairment.

Outpatient clinic visits within 1–5 years after diagnosis of meningitis were more frequent in children with a history of bacterial meningitis than in children in the comparison cohort (297 [66·0%] *vs* 1748 [36·8%]). A similar pattern was seen for hospital admissions: 175 (38·9%) children in the cohort with a history of bacterial meningitis were admitted within 1–5 years after diagnosis, compared with 1324 (27·9%) of children in the comparison cohort (appendix p 18). Negative binomial regression confirmed higher rates of outpatient clinic visits (rate ratio 4·5 [95% CI 3·9–5·2]) and hospital admissions (4·1 [3·6–4·7]) in the first 10 years after disease onset in the cohort with a history of bacterial meningitis than in the comparison cohort. Similar associations were observed in pathogen-specific analyses (results not shown). In both countries, household income was similar in families of the cohort with a history of bacterial meningitis and of the comparison cohort (appendix p 19). Household income remained similar when stratifying the cohorts with a history of bacterial meningitis by diagnosis of NDIs or death at any age during follow-up (appendix p 20). In the Netherlands, lower median incomes were observed in families of children with a history of bacterial meningitis who had died or been diagnosed with any NDI than in families of children with a history of bacterial meningitis who had survived and did not have any NDIs, with substantial overlap between the distributions.

## Discussion

In this nationwide cohort study in Denmark and the Netherlands, we assessed short-term and long-term outcomes following bacterial meningitis during infancy in a large dataset of 2216 children with a history of meningitis matched to 22 127 children without a history of bacterial meningitis. Risk of mortality in children with a history of bacterial meningitis differed between causative pathogens. In the Netherlands, children with a history of *S agalactiae* meningitis had the highest risk of mortality, followed by those with a history of *E coli* meningitis. In Denmark, children with a history of *H influenzae* or *E coli* meningitis had the highest risks of mortality. Risk of death in the first 3 months after meningitis onset was consistent with case fatality rates reported from studies of bacterial meningitis in young children in Finland, France, Ireland, the UK, and the USA.^25^, ^26^, ^27^, ^28^ Notably, in this study, individuals who had survived bacterial meningitis during infancy remained at increased risk of premature death during follow-up into adolescence, substantiating findings from previous studies with other designs, conducted in other regions and study populations (eg, adults or older children [aged 1–18 years]).^8^, ^9^, ^10^ This finding could be partially explained by the onset of NDIs during childhood that lead to an increased risk of mortality, as was suggested by a systematic review.^29^ Risk of mortality was lower in Denmark than in the Netherlands, which could be due to differences in case definition when diagnosing bacterial meningitis. In Denmark, diagnoses were based on ICD-10 codes; therefore, some patients without a positive CSF culture are likely to have been included in the bacterial meningitis cohort. Consistent with this explanation, a retrospective cohort study in the USA showed lower case fatality rates in patients with negative CSF cultures than in those with positive CSF cultures.^30^ We note that, given the seriousness of meningitis, antibiotics are sometimes given before lumbar puncture, especially in clinically unstable children, resulting in negative CSF cultures in some cases of bacterial meningitis.

Our study highlights the importance of including a comparison cohort. In addition to quantifying absolute risks, we compared risk of mortality between children with and without a history of bacterial meningitis. In our analyses, pathogens associated with the highest risk of death were not necessarily those associated with the highest relative increase in risk of mortality at the typical age of disease onset. A possible reason is that risk of death due to any cause is not evenly distributed during infancy.^31^ In the Netherlands, half of infant mortality occurs in the first week of life, and three-quarters occur within the first month.^21^ Therefore, matching by age at disease onset resulted in a lower rate of mortality among children in the comparison cohort who were matched to patients with a later onset of meningitis. This explains why risk of death was higher in children with bacterial meningitis caused by a pathogen that causes neonatal meningitis (eg, *E coli* and *S agalactiae*), whereas hazard ratios were higher for pathogens that cause disease after the neonatal period (eg, *S pneumoniae* and *N meningitidis*). Furthermore, in both countries, mortality in children who had developed pneumococcal meningitis was higher than in children with a history of meningococcal meningitis, despite similar age distributions. This finding is consistent with those of previous studies.^28^, ^32^, ^33^

Risk of NDIs in children with a history of bacterial meningitis increased during childhood. This association might be explained by the concept of so-called growing into deficits, in which pre-existing impairments become more apparent with increasing social and educational demands, as children grow.^34^ The difference in risk of NDIs between the cohort with a history of bacterial meningitis and the comparison cohort was highest after 5 years of follow-up, possibly because children with a history of bacterial meningitis developed NDIs relatively early in life or presented with more severe, and consequently more easily diagnosable, impairments. Alternative explanations include differences between these groups in affected domains, the possibility that parents or caregivers of children with a history of bacterial meningitis are more alert to developmental problems, or children who have survived bacterial meningitis receive more intense medical follow-up, compared with those without a history of meningitis. In line with our study, a retrospective cohort study of patients aged 1 month to 15 years with bacterial meningitis in Denmark concluded that neurological sequelae were frequent after bacterial meningitis.^35^ In our study, compared with children unexposed to meningitis, infants who survived *S agalactiae* or pneumococcal meningitis were at the highest risk of moderate or severe NDIs during childhood. This finding supports the results from a large meta-analysis that did not include neonatal cases.^12^ We found that the RRs for needing educational support were highest after pneumococcal meningitis, consistent with a population-based cohort study in Denmark that described the achievements of adults who were diagnosed with bacterial meningitis during childhood (aged <12 years) compared with their siblings and a comparison cohort.^36^

Additionally, bacterial meningitis in infants was associated with increased health-care use throughout childhood. Future work using similar large-scale administrative datasets should estimate the cost of this increased health-care use and assess the proportion that represents appropriate clinical follow-up for these children, as well as the other medical conditions that are more common after bacterial meningitis.

Despite the impact of acute illness with bacterial meningitis, the risk of severe sequelae, and increased health-care use, we found little effect of the disease on household income in our descriptive analysis. This pattern might be explained by the welfare systems of Denmark and the Netherlands. Health care is largely financed through general taxation, and a study in Denmark found that adults with a history of bacterial meningitis received more disability pensions than did the general population.^36^ A similar pattern might not be present in other settings. In low-income and middle-income countries, where most of the burden of meningitis occurs, it is likely that this condition has important effects on family-level income, either due to hospitalisation for the acute episode or long-term care for a child with NDIs.^37^ Of note, data on family expenditure were not available, so we were not able to assess whether bacterial meningitis increased expenditure.

Our study has several limitations. First, age of meningitis onset might have been incorrectly estimated in Denmark for children with a history of *E coli* or *S agalactiae* meningitis, given that it was based on date of hospital admission. However, the distribution of age at onset was similar to findings from the Netherlands. Second, risk of NDIs might have been underestimated because only cases severe enough to prompt a clinical visit or hospital admission in Denmark or to require educational support in the Netherlands were identified. Additionally, post-discharge mortality could have been higher in children with severe neurological impairments and these children might have died before a diagnosis of NDI could be made. Furthermore, the approach used in analyses on health-care use might underestimate usage because children who died at an earlier time, either in hospital or following discharge, would not have contributed to the health-care estimates.

The strengths of our study include long follow-up, a large sample size, and a large comparison cohort. The novelty of our work is evident in three areas: use of two different study settings with complementary case definitions that cover the case mix of clinical practice, examination of outcomes for different pathogens in the same population, and use of two different types of definitions for NDI. ICD-10 codes were used to define NDI in Denmark, while a functional outcome was used in the Netherlands, which assessed the need for educational support or special education. The similar pattern of results in the two settings support the robustness of our findings, paralleling results of studies in older children. Furthermore, we attempted to minimise bias by matching the comparison cohorts on sex and age and by performing sensitivity analyses with additional adjustment for gestational age.

In conclusion, despite reduced incidence of bacterial meningitis over recent decades, the disease has profound effects throughout the life course on infants who develop this condition. Our results underline the public health importance of vaccines that prevent bacterial meningitis and the need to promote, increase, and sustain vaccine coverage to reduce the associated burden. Our data quantify the long-term consequences of bacterial meningitis in infants, facilitating estimation of the relative contributions of bacterial pathogens to the total burden of meningitis in Denmark, the Netherlands, and other settings with similar epidemiological resources and health-care systems. Future analytical work could use administrative and medical record databases to compare outcomes in children receiving different care during and after their acute illness, to generate observational evidence for improved care, and to inform future trials. Importantly, our findings show that it is imperative to provide long-term support for families of children with a history of meningitis.

## Data sharing

Although data protection regulations in Denmark and the Netherlands do not allow sharing of individual participant data, datasets with selected aggregated data are available upon reasonable request. Proposals should be directed to MWB. Individuals who request data will be asked to sign a data access agreement.

## Declaration of interests

We declare no competing interests.
