# Supplementary material for: Short-term and long-term risk of mortality and neurodevelopmental impairments after bacterial meningitis during infancy in children in Denmark and the Netherlands: a nationwide matched cohort study
Source: Lancet Child Adolesc Health. 2022 Sep;6(9):633–42. doi: 10.1016/S2352-4642(22)00155-9 (PMC9365703; doi:10.1016/S2352-4642(22)00155-9)
Supplement: Supplementary appendix [file mmc1.pdf]

THE LANCET
Child & Adolescent Health

Supplementary appendix

This appendix formed part of the original submission and has been peer reviewed. We post it as supplied by the authors.

Supplement to: Snoek L, Gonçalves BP, Horváth-Puhó E, et al. Short-term and long-term risk of mortality and neurodevelopmental impairments after bacterial meningitis during infancy in children in Denmark and the Netherlands: a nationwide matched cohort study. *Lancet Child Adolesc Health* 2022; published online July 4. https://doi.org/10.1016/S2352-4642(22)00155-9.

SUPPLEMENTARY MATERIAL

Paper Title: Short-term and long-term risk of mortality and neurodevelopmental impairments after bacterial meningitis during infancy in children in Denmark and the Netherlands: a nationwide matched cohort study

Authors: Linde Snoek[*1,2], Bronner P Gonçalves[*3,4], Erzsébet Horváth-Puhó[*5], Merel N van Kassel[1,2], Simon R Procter[3,4], Kirstine K Søgaard[5,6], Jaya Chandna[3,4], Arie van der Ende[7,8], Diederik van de Beek[1,2], Matthijs C Brouwer[1,2], Henrik T Sørensen[5], Joy E Lawn[3,4], Merijn W Bijlsma[2,8]

[1] Department of Neurology, Amsterdam Neuroscience, Amsterdam University Medical Centre, University of Amsterdam, Amsterdam, Netherlands

[2] Amsterdam Neuroscience, Neuroinfection and Inflammation, Amsterdam, Netherlands

[3] Maternal, Adolescent, Reproductive & Child Health (MARCH) Centre, London School of Hygiene & Tropical Medicine, London, United Kingdom

[4] Department of Infectious Disease Epidemiology, London School of Hygiene & Tropical Medicine, London, United Kingdom

[5] Department of Clinical Epidemiology, Aarhus University Hospital and Aarhus University, Aarhus, Denmark

[6] Department of Clinical Microbiology, Aalborg University Hospital and Aalborg University, Aalborg, Denmark

[7] Department of Medical Microbiology and Infection Prevention, Amsterdam Infection and Immunity, Amsterdam University Medical Centre, University of Amsterdam, Amsterdam, Netherlands

[8] Netherlands Reference Laboratory for Bacterial Meningitis, Amsterdam University Medical Centre, University of Amsterdam, Amsterdam, Netherlands

[9] Department of Paediatrics, Amsterdam University Medical Centre, University of Amsterdam, Amsterdam, Netherlands

*Joint first authors: Linde Snoek[1], Bronner P Gonçalves[2,3], Erzsébet Horváth-Puhó[4]

Contents

STROBE checklist (page 3-4)

Supplementary Methods (pages 5 – 7)
Additional information on databases (Denmark).
Additional information on databases (the Netherlands).
Additional information on the inclusion of children for neurodevelopmental impairment analyses (the Netherlands).

Supplementary Tables (pages 8 – 17)
Supplementary Table S1 - Health registers and variables used in the study.
Supplementary Table S2 - Median gestational age per causative pathogen.
Supplementary Table S3 - Mortality risks and HRs for children with bacterial meningitis and comparison cohort members, with adjustment for gestational age.
Supplementary Table S4 - Mortality risks and HRs for children with bacterial meningitis and comparison cohort members, among those who survived the first month.
Supplementary Table S5 - NDI outcomes for children with bacterial meningitis and comparison cohort members, with adjustment for gestational age.
Supplementary Table S6 – Denmark: NDI outcomes for any domain or domain-specific need.

Supplementary Figures (pages 18-20)
Supplementary Figure S1 - Health-care use among children with bacterial meningitis (red bars) and among children in the comparison cohort (blue bars).
Supplementary Figure S2 - Household income for families of children with bacterial meningitis (red bars) and for families of children without bacterial meningitis (blue bars).
Supplementary Figure S3 - Household income for families of children with bacterial meningitis (red bars) and for families of comparison cohort members (blue bars), stratified by NDI diagnosis or death at any age.

References (page 21)

STROBE checklist.

Our study is reported according to the Strengthening the Reporting of Observational Studies in Epidemiology (STROBE) statement. The STROBE checklist, downloaded from https://www.strobe-statement.org, is shown below. For items 7 and 8 (related to diagnostic criteria and comparability of assessment methods), relevant information is included both in the *Methods* section of the main text and in **Supplementary Table S1**.

	**Item**	**Recommendation**	**Reported on manuscript page**
**Title and abstract**	1	(*a*) Indicate the study's design with a commonly used term in the title or the abstract	1
		(*b*) Provide in the abstract an informative and balanced summary of what was done and what was found	1
**Introduction**			
Background/ rationale	2	Explain the scientific background and rationale for the investigation being reported	1-2
Objectives	3	State specific objectives, including any prespecified hypotheses	2
**Methods**			
Study design	4	Present key elements of study design early in the paper	2-3
Setting	5	Describe the setting, locations, and relevant dates, including periods of recruitment, exposure, follow-up, and data collection	2-3
Participants	6	(*a*) Give the eligibility criteria, and the sources and methods of selection of participants. Describe methods of follow-up	2-3
		(*b*) For matched studies, give matching criteria and number of exposed and unexposed	3
Variables	7	Clearly define all outcomes, exposures, predictors, potential confounders, and effect modifiers. Give diagnostic criteria, if applicable	2-3
Data sources/ measurement	8	For each variable of interest, give sources of data and details of methods of assessment (measurement). Describe comparability of assessment methods if there is more than one group	2-3
Bias	9	Describe any efforts to address potential sources of bias	4, 9
Study size	10	Explain how the study size was arrived at	2-3
Quantitative variables	11	Explain how quantitative variables were handled in the analyses. If applicable, describe which groupings were chosen and why	3-4
Statistical methods	12	(*a*) Describe all statistical methods, including those used to control for confounding	3-4
		(*b*) Describe any methods used to examine subgroups and interactions	3-4
		(*c*) Explain how missing data were addressed	3-4
		(*d*) If applicable, explain how loss to follow-up was addressed	3
		(*e*) Describe any sensitivity analyses	4
**Results**			
Participants	13	(a) Report numbers of individuals at each stage of study—eg numbers potentially eligible, examined for eligibility, confirmed eligible, included in the study, completing follow-up, and analysed	4-5, Fig 1
		(b) Give reasons for non-participation at each stage	Fig 1
		(c) Consider use of a flow diagram	Fig 1

Descriptive data	14	(a) Give characteristics of study participants (eg demographic, clinical, social) and information on exposures and potential confounders	4-5
		(b) Indicate number of participants with missing data for each variable of interest	Table 1, Supplementary appendix
		(c) Summarise follow-up time (eg, average and total amount)	5
Outcome data	15	Report numbers of outcome events or summary measures over time	5-7
Main results	16	(*a*) Give unadjusted estimates and, if applicable, confounder-adjusted estimates and their precision (eg, 95% confidence interval). Make clear which confounders were adjusted for and why they were included	5-7, Supplementary appendix
		(*b*) Report category boundaries when continuous variables were categorized	Table 1, Table S3, Table S5
		(*c*) If relevant, consider translating estimates of relative risk into absolute risk for a meaningful time period	-
Other analyses	17	Report other analyses done—eg analyses of subgroups and interactions, and sensitivity analyses	5-7
**Discussion**			
Key results	18	Summarise key results with reference to study objectives	7-9
Limitations	19	Discuss limitations of the study, taking into account sources of potential bias or imprecision. Discuss both direction and magnitude of any potential bias	9
Interpretation	20	Give a cautious overall interpretation of results considering objectives, limitations, multiplicity of analyses, results from similar studies, and other relevant evidence	7-9
Generalisability	21	Discuss the generalisability (external validity) of the study results	7-9
**Other information**			
Funding	22	Give the source of funding and the role of the funders for the present study and, if applicable, for the original study on which the present article is based	1

*Give information separately for cases and controls in case-control studies and, if applicable, for exposed and unexposed groups in cohort and cross-sectional studies.

Supplementary Methods

Additional information on national databases

In this section of the Supplementary Appendix, we provide additional information on the national databases that were used both to assess exposure, outcomes (mortality, neurodevelopment impairments, household income, and health care utilisation) and covariates.

Note: In the two countries where this study was performed, a risk-based intrapartum antibiotic prophylaxis policy was adopted nearly two decades ago. However, data are not available on its use for individual infants.

Denmark

The Danish National Health Service provides tax-supported health care. Individual-level linkage of all registries is possible using the unique personal identification number assigned to all Danish residents at birth or upon immigration. Due to Danish data protection regulations, only results describing at least 5 people could be reported.

In our analyses, the following databases were used:

- The **Danish Civil Registration System** is an administrative register established in 1968. This registry contains individual-level information on all persons residing in Denmark and provides daily updates on vital statistics, including dates of birth, migration, emigration, and death. Upon registration in the Civil Registration System, each Danish resident receives a unique ten-digit identification number (CPR number) allowing unambiguous individual-level record linkage among different Danish registers. When analyses were performed (2020), data from the Danish Civil Registration System were available up to 31/12/2018.

- The **Danish Medical Birth Register** was established in 1973 based on paper birth forms. It includes prospectively collected data on all deliveries in Denmark. Major changes in the construction and content of the Medical Birth Registry were implemented in 1997, when electronic registration of births replaced paper forms. The Medical Birth Registry contains information on the index pregnancy, pregnancy-related characteristics of the mother, details of the delivery (*e.g.*, date of delivery, caesarean section), and outcome characteristics of the newborn.[1]

- The **Danish National Patient Registry** contains information on all admissions to Danish non-psychiatric hospitals since 1977 and on outpatient clinic visits and emergency room visits since 1995. Each hospital discharge or outpatient clinic visit is recorded with one primary diagnosis and one or more secondary diagnoses classified according to the International Classification of Diseases, Eighth Revision through 1993 and Tenth Revision thereafter. The National Patient Registry also contains information on examinations, certain inpatient medical treatments, and surgical procedures.[2] When analyses were performed (2020), data from the National Patient Registry were available up to 31/12/2018.

- The **Danish Psychiatric Central Research Registry** contains information on psychiatric hospital admissions starting in 1969. In 1995, data were added on outpatient clinic treatment and emergency room contacts for psychiatric conditions. The register contains the following information: CPR number, dates of any admission and discharge or start and end time of any outpatient treatment including emergency room visits; all diagnoses; type of referral; place of treatment with identification of the specific department; municipality of residence; and mode of admission (acute or planned).[3]

- The **Income Statistics Register** contains high-quality data on the income composition of the Danish population, including variables related to individual income (*e.g.*, salary, income from private pensions). Data are available from 1980 onwards.[4] Gross income is measured before deduction of labor market and special pension contributions. Income values are available in Danish Krone in the Income Statistics Register. In this study, income values were converted from Krone to Euros.[5] Annual household incomes were defined as the sum of the gross income of the cohort member's parents.

The Netherlands

The Dutch Healthcare System is a social welfare-based system, providing mandatory health care insurance for all residents aged 18 years or older. The System provides a standard, nearly comprehensive, benefit package as specified by law. Due to Dutch data protection regulations, only results describing at least 10 people could be reported.

The following databases were used in this cohort study:

- The Netherlands Reference Laboratory for Bacterial Meningitis database (NRLBM): the NRLBM receives approximately 90% of CSF isolates from Dutch patients with suspected bacterial meningitis.[6] The NRLBM also receives data on some basic patient characteristics including date of birth, sex, residency, and culture date. We used the culture date to calculate the age of meningitis onset. When the culture date was not available (12·9%), we used the date the isolate was sent to or received by the NRLBM.

  The NRLBM dataset was transferred to the secure environment of Statistics Netherlands and linked to unique personal registry numbers based on date of birth, sex, and residency.[7] Infants with no or multiple possible unique personal registry numbers were linked using hospital data from the PeriNed perinatal registry and the Dutch Hospital Data Registry.[8]

- PeriNed: The Perinatal Registry has covered over 97% of all births in the Netherlands since 2000.[8, 9] This database includes variables related to the mother (age, obstetric history), pregnancy (mode of delivery), birth (birth weight, gestational age, Apgar score) and neonatal (re)admission up to 28 days of age. These data are obtained from the four professional organizations of midwives, gynaecologists, general practitioners, and paediatricians involved in childbirth care: the KNOV (Royal Dutch Organization of Obstetricians), the LHV (National General Practitioners Association), the NVOG (Dutch Association for Obstetrics and Gynaecology) and the NVK (Dutch Association for Paediatrics).

- Statistics Netherlands: Statistics Netherlands is a Dutch governmental institution that contains a large number of population registry datasets with individual-level information related to health, well-being, income and education.[7] Each Dutch citizen is assigned a unique personal registry number at birth, which can be used to link this individual-level data. Datasets are available for researchers under strict conditions. (Further information is available at microdata@cbs.nl). All results presented in this paper are based on analyses that were performed by researchers affiliated with the Amsterdam University Medical Center, using the following non-public datasets:

  - Dutch Hospital data registries: Statistics Netherlands maintains non-public registries containing hospital admission dates, discharge dates, diagnoses, and information on treatment and other inpatient procedures. For the current study, these datasets were used solely to link bacterial meningitis patients to the correct personal registry number.

  - Municipal Personal Records Database: this database records all deaths with dates in the Netherlands. It contains information from 1995 onwards, and is updated every year.

  - Dutch Primary School Registry and Dutch Special Education School Registry: these national databases were used to ascertain the number of children needing additional support in a regular school or education in a special needs school. These datasets contain information from 2008 onwards. To handle missing data for children for whom follow-up started at an age older than five years (e.g., a child born in 2000 was already 8 years old in 2008), we assumed that educational needs at the older age were the same as during preceding years.

  - Dutch Income Panel Survey*:* this national database, established in 2003, contains information on household income for every Dutch household for each calendar year starting on January 1st. Using the personal registry number, children can be linked to their respective households starting at age 1.

  When analyses were performed (2020), data from the Municipal Personal Records Database, the Dutch Primary School Registry and Dutch Special Education School Registry were available up to 31/12/2019.

Additional information on the inclusion of children in analyses for neurodevelopmental impairment (NDI)

The Netherlands

Among 1,646 infants with a history of bacterial meningitis, 1,394 (84·7%) reached the required age of 5 years (116 infants died and 136 did not reach the age of 5 years). Of these 1,394 infants, 102 (7·3%) were born too early and were therefore not registered in the education databases (2008-2019). For 1,220 (94·4%) of the remaining 1,292 infants, information on education was available. For 10-year NDI analyses, 1,190 (72·3%) of 1,646 infants reached the required age (127 infants died and 329 had not yet reached the age of 10 years). For 100 (8·4%) of the 1,190 infants, educational data was not available because they were born too early. For 1,036 (95·0%) of the remaining 1,090 infants, educational information was available.

Supplementary Tables

Table S1. Health Registers and variables used in the study.

	**Denmark**	**The Netherlands**
**Exposures**		
**Bacterial meningitis**	**Danish National Patient Registry**<br>*(Includes inpatients and outpatients, primary and secondary discharge diagnoses)*	**Netherlands Reference Laboratory for Bacterial Meningitis**
	ICD-10 codes ≤ 365 days after birth:<br>· *Streptococcus agalactiae*:<br>{G00.2 - Streptococcal meningitis<br>OR<br>[(P36.0 - Sepsis of newborn due to GBS OR<br>A40.1 - Sepsis due to GBS)<br>AND<br>(G00.9 - Bacterial meningitis, unspecified OR<br>G03.9 - Meningitis, unspecified)]}<br>AND<br>No concurrent ICD-10 codes: A400, A402, A403<br>· *Streptococcus pneumoniae*: G00.1<br>· *Neisseria meningitidis*: A39.0<br>· *Escherichia coli*: G00.8B<br>· *Haemophilus influenzae*: G00.0	Positive CSF culture for bacterial pathogen.
**Outcomes**		

**Mortality**	**Danish Civil Registration System**<br>(*updated daily*)	**Statistics Netherlands:**<br>· **Municipal Personal Records Database**<br>(*Available for 1995-2020; updated yearly*)
	Classification<br>· Death during acute phase: died <3 months after disease onset<br>· 5-year mortality: died ≤ 5 years after disease onset<br>· 10-year mortality: died ≤ 10 years after disease onset	Classification<br>· Death during acute phase: died <90 days after disease onset<br>· 5-year mortality: died ≤ 5 years after disease onset<br>· 10-year mortality: died ≤ 10 years after disease onset
**Neurodevelopment impairment**	**Danish National Patient Registry and Danish Psychiatric Central Research Register**<br>*(Includes inpatients and outpatients, and primary and secondary discharge diagnoses*)	**Statistics Netherlands:**<br>· **Dutch Primary School Registry**<br>*(available for 2008-2020*)<br>· **Dutch Special Education School Registry**<br>*(available for 2008-2020*)
Any	Any of the domain specific ICD-10 codes listed below:<br>· Mild: One or two domain-specific mild codes<br>· Moderate: Three or more mild codes **OR** one moderate code<br>· Severe: Two or more moderate codes **OR** at least one severe code	Based on labels in registry files<br>· Mild: Label for special education need in Dutch Primary School Registry = attending regular school with additional support<br>· Moderate-Severe: Label for special education in Dutch Special Education School Registry = attending special school
Motor	Any of the ICD-10 codes listed below:<br>· Mild: F82, R27.0, R27.8, R26.0, R26.1, G24.9, G25.9 (developmental disorder of motor function, ataxia, other lack of coordination, ataxic or paralytic gait, dystonia, extrapyramidal and movement disorder)	N.A.

	· Moderate: G80.1, G80.3, G80.4, G80.8, G80.9 (spastic diplegic cerebral palsy, dyskinetic, ataxic or other cerebral palsy)<br>· Severe: G80.0 (Spastic quadriplegic cerebral palsy)	
Hearing	Any of the ICD-10 codes listed below:<br>· Mild: H90.1, H90.4, H90.7 (unilateral hearing loss with unrestricted hearing on contralateral side [conductive and/or sensorineural])<br>· Severe: H90.0, H90.3, H90.6 (bilateral hearing loss [conductive and/or sensorineural])<br>· Not categorized: H90.2, H90.5, H90.8 (unspecified hearing loss [conductive and/or sensorineural])	N.A.
Vision	Any of the ICD-10 codes listed below:<br>· Mild: H53.0, H53.1, H53.2, H53.4, H54.4, H54.5, H54.6 (amblyopia ex anopsia, subjective visual disturbances, diplopia, visual field defects, blindness [monocular], moderate or severe visual impairment [monocular])<br>· Moderate: H54.2 (moderate visual impairment [binocular])<br>· Severe: H54.0, H54.1 (blindness [binocular], severe visual impairment [binocular])	N.A.
Cognitive	Any of the ICD-10 codes listed below:<br>· Mild: F70, F80.0, F80.1, F80.2, F80.9, F81.0, F81.1, F81.2 (mild mental retardation [MR], speech articulation disorder, expressive or receptive language disorder, other disorders of speech and language,	N.A.

	reading or spelling disorder, disorder of arithmetic skills)<br>· Moderate: F71, F81.3, F83, F84.0, F84.1, F84.3, F84.5 (moderate MR, mixed disorder of scholastic skills, mixed developmental disorders, Autistic disorder, atypical autism, disintegrative disorder, Asperger syndrome)<br>· Severe: F72, F73, F80.3, F84.2, F84.4 (severe or profound MR, acquired aphasia with epilepsy, Rett's syndrome, overactive disorder with MR and stereotypic movement)<br>· Not categorized: F78, F79, F84.8, F84.9, F88 (other MR, other pervasive developmental disorder, other disorders of psychological development)	
Social/behavioral	Any of the ICD-10 codes listed below:<br><br>Mild: F90.0, F90.1, F90.2, F90.8, F90.9, F91.0, F91.1, F91.2, F91.3, F91.8, F91.9, F92.0, F92.8, F92.9, F93.0, F93.1, F93.2, F93.3, F93.8, F93.9, F94.1, F94.2, F94.8, F94.9, F95.1, F95.2, F95.8, F95.9, F98.0, F98.1, F98.2, F98.3 (attention-deficit hyperactivity disorders [predominantly inattentive, hyperactive, combined or other type], conduct disorder [confined to family context, childhood or adolescent-onset type, other], oppositional defiant disorder, other conduct disorders, emotional disorders with onset specific to childhood, disorders of social functioning, Tic disorder, other behavioral and emotional disorders)	N.A.

Multi-domain	If child has more than one affected domain AND<br>· Mild: Two domains mildly affected<br>· Moderate: Three or more domains mildly affected, **OR** one moderately affected and at least one mildly affected<br>· Severe: Two or more domains moderately affected, **OR** one domain severely affected and at least one domain mildly or moderately affected, OR two or more domains severely affected	N.A.

N.A.: not available for the Netherlands.

Table S2. Median gestational age per causative pathogen.

	**Denmark**<br>**n = 570**	**The Netherlands**<br>**n=1646**
**Causative pathogen**	Gestational age in weeks (IQR)	Gestational age in weeks (IQR)
S. agalactiae	39+0 (36+5 - 40+3)	39+2 (37+3 - 40+4)
S. pneumoniae	40+0 (38+5 - 40+6)	39+4 (38+2 - 40+5)
N. meningitidis	39+6 (38+1 - 41+0)	39+4 (38+4 - 40+6)
E.coli	34+4 (32+2 - 38+3)	38+4 (33+3 - 39+6)
H. influenzae	40+2 (39+6 - 41+3)	40+1 (39+2 - 41+0)
*Other**	N.A.	38+1 (31+2 - 40+2)

*The group of 'other' bacteria consists of less common, gram-positive and gram-negative bacteria. Due to Dutch data protection regulations, we are not allowed to provide detailed information on these causative pathogens. Abbreviations: N.A. = not available.

Table S3. Mortality of children with bacterial meningitis and comparison cohort members, with adjustment for gestational age.

	**Denmark**	**The Netherlands**
	**HR (95% CI) with adjustment for GA**	**HR (95% CI) with adjustment for GA**
**Time since disease onset**	**All pathogens**	
**<3 months**	-	41·8 (21·0 - 82·9)
**<5 years**	52·7 (20·0 - 138·5)	21·3 (13·3 - 34·0)
**<10 years**	44·0 (17·9 - 108·1)	16·3 (10·5 - 25·3)
	S. agalactiae	
**<3 months**	-	20·3 (8·3 - 49·7)
**<5 years**	100·3 (12·5 - 805·6)	13·7 (6·2 - 30·4)
**<10 years**	100·3 (12·5 - 805·6)	12·9 (6·1 - 27·4)
	S. pneumoniae	
**<3 months**	-	-
**<5 years**	81·8 (10·2 - 654·8)	31·4 (13·4 - 73·4)
**<10 years**	41·1 (8·7 - 193·9)	26·8 (12·0 - 60·1)
	N. meningitidis	
**<3 months**	NA	75·3 (9·4 - 603·2)
**<5 years**	16·6 (2·8 - 99·4)	20·7 (6·4 - 67·5)
**<10 years**	16·6 (2·8 - 99·4)	13·0 (4·6 - 36·9)
	H. influenzae	
**<3 months**	-	-
**<5 years**	-	-
**<10 years**	-	7·0 (0·4 - 133·3)
	E. coli	
**<3 months**	-	21·0 (2·3 - 195·0)
**<5 years**	33·6 (2·6 - 439·4)	12·0 (2·9 - 49·9)
**<10 years**	33·6 (2·6 - 439·4)	8·1 (1·8 - 37·5)

Mortality is expressed as time since onset of disease. HRs were calculated with adjustment for matching factors and gestational age (two categories: <37 weeks and ≥37 weeks).
Abbreviations: BM=bacterial meningitis, HR=hazard ratio, GA=gestational age

Table S4. Mortality risks and HRs for children with bacterial meningitis and comparison cohort members, among those who survived the first month.

	**Denmark**			**The Netherlands**		
	**BM cohort Mortality risk (95% CI)**	**Comparison cohort Mortality risk (95% CI)**	**HR (95% CI)**	**BM cohort Mortality risk (95% CI)**	**Comparison cohort Mortality risk (95% CI)**	**HR (95% CI)**
**Time since onset**	**All pathogens**					
**<1 month**	3·5 (2·3 - 5·4)	-	-	5·4 (4·3 - 6·4)	0·1 (0·0 - 0·1)	87·9 (45·7 - 169·2)
**1 months-10 years**	1·5 (0·8 - 3·0)	0·1 (0·1 - 0·3)	13·9 (4·8 - 40·1)	3·4 (2·5 - 4·5)	0·3 (0·2 - 0·4)	10·3 (6·7 - 15·8)

Mortality is expressed as time since onset of disease. HRs are adjusted for matching variables (i.e., sex and year of birth).

Abbreviations: BM=bacterial meningitis; HR=hazard ratio

Table S5. NDI outcomes for children with bacterial meningitis and for comparison cohort members, with adjustment for gestational age.

A. Denmark

	**All pathogens**		***S. agalactiae***		***S. pneumoniae***		***N. meningitidis***	
	**Any**	**Moderate/ severe**	**Any**	**Moderate/ severe**	**Any**	**Moderate/ severe**	**Any**	**Moderate/ severe**
**Age**	RR (95% CI)	RR (95% CI)	RR (95% CI)	RR (95% CI)	RR (95% CI)	RR (95% CI)	RR (95% CI)	RR (95% CI)
**<5y**	7·3 (5·0 - 10·8)	11·7 (7·1 - 19·2)	11·0 (5·3 - 23·1)	14·9 (5·1 - 43·3)	7·4 (4·2 - 12·9)	12·5 (6·2 - 25·2)	1·8 (0·7 - 4·4)	2·9 (0·8 - 10·9)
**<7y**	5·0 (3·6 - 7·1)	6·9 (4·5 - 10·7)	5·4 (2·9 - 10·4)	8·4 (3·5 - 20·1)	5·6 (3·5 - 8·8)	7·6 (4·3 - 13·2)	1·5 (0·7 - 3·4)	1·1 (0·3 - 4·8)
**<10y**	3·4 (2·4 - 4·8)	4·9 (3·2 - 7·4)	4·4 (2·6 - 7·6)	6·5 (3·0 - 13·8)	2·9 (1·8 - 4·6)	4·7 (2·6 - 8·2)	1·7 (0·9 - 3·3)	2·1 (0·9 - 5·0)
**<15y**	2·7 (1·8 - 4·0)	3·7 (2·4 - 5·8)	3·3 (1·9 - 5·9)	5·0 (2·4 - 10·3)	2·0 (1·2 - 3·3)	2·8 (1·5 - 5·1)	1·9 (1·0 - 3·6)	2·0 (0·9 - 4·4)
**<20y**	2·9 (1·4 - 6·0)	3·8 (1·6 - 9·0)	2·1 (0·5 - 8·7)	3·8 (0·8 - 17·8)	1·9 (0·7 - 5·3)	N.A.	3·4 (1·3 - 8·4)	3·2 (1·0 - 10·2)

B. Netherlands

	**All pathogens**		***S. agalactiae***		***S. pneumoniae***		***N. meningitidis***	
	**Any**	**Moderate/ severe**	**Any**	**Moderate/ severe**	**Any**	**Moderate/ severe**	**Any**	**Moderate/ severe**
**Age**	RR (95% CI)	RR (95% CI)	RR (95% CI)	RR (95% CI)	RR (95% CI)	RR (95% CI)	RR (95% CI)	RR (95% CI)
**<5y**	6·3 (4·2 - 9·3)	7·2 (4·6 - 11·1)	4·0 (1·8 - 8·9)	3·8 (1·6 - 9·1)	9·4 (5·5 - 16·2)	12·8 (6·9 - 23·6)	N.A.	N.A.
**<7y**	3·3 (2·5 - 4·3)	4·7 (3·4 - 6·3)	2·7 (1·5 - 4·6)	3·4 (1·8 - 6·4)	3·6 (2·4 - 5·4)	5·8 (3·7 - 9·0)	N.A.	N.A.
**<10y**	2·5 (2·0 - 3·0)	3·9 (3·0 - 5·1)	2·7 (1·8 - 4·3)	3·1 (1·7 - 5·7)	2·2 (1·6 - 3·1)	4·2 (2·9 - 6·2)	2·5 (1·6 - 3·8)	3·4 (1·9 - 6·2)
**<11y**	2·3 (1·9 - 2·8)	4·0 (3·1 - 5·2)	2·7 (1·7 - 4·1)	3·2 (1·7 - 6·1)	2·2 (1·6 - 3·0)	4·6 (3·2 - 6·7)	2·3 (1·5 - 3·4)	3·3 (1·9 - 6·0)

Risk ratios for the association between history of bacterial meningitis and NDI/education outcomes were estimated using a modified Poisson regression model, with adjustment for matching factors and gestational age (two categories: <37 weeks and ≥37 weeks).
Abbreviation: RR=risk ratio.

Table S6. Denmark: NDI outcomes for any domain or domain-specific need.

	Any domain		*Domain specific*					*Multi domain*	
			**Cognitive**	**Motor**	**Social/ behavioural**	**Hearing**	**Vision**		
	**Any**	**Moderate/ severe**	**Any**	**Any**	**Any**	**Any**	**Any**	**Any**	**Moderate/ severe**
**Age**	RR (95% CI)	RR (95% CI)	RR (95% CI)	RR (95% CI)	RR (95% CI)	RR (95% CI)	RR (95% CI)	RR (95% CI)	RR (95% CI)
**<5y**	6·3 (4·4 - 9·0)	9·5 (5·9 - 15·2)	4·3 (2·3 - 7·9)	16·0 (7·8 - 32·8)	4·0 (2·0 - 7·9)	22·4 (12·0 - 41·7)	10·1 (3·3 - 31·2)	28·3 (10·3 - 78·2)	30·4 (9·9 - 93·6)
**<7y**	3·8 (2·8 - 5·1)	6·0 (3·8 - 9·3)	2·5 (1·4 - 4·5)	13·5 (6·4 - 28·4)	1·8 (1·1 - 3·1)	28·1 (15·0 - 52·4)	8·7 (2·9 - 25·7)	4·8 (2·3 - 10·2)	4·5 (2·0 - 10·3)
**<10y**	2·5 (1·9 - 3·5)	3·5 (2·2 - 5·4)	1·8 (1·0 - 3·1)	10·2 (4·8 - 21·7)	1·7 (1·1 - 2·7)	18·9 (10·2 - 35·0)	6·1 (2·2 - 16·6)	4·4 (2·3 - 8·3)	3·2 (1·5 - 6·8)
**<15y**	2·2 (1·6 - 3·2)	3·1 (2·0 - 4·9)	1·5 (0·8 - 2·8)	14·5 (5·6 - 37·6)	1·4 (0·8 - 2·4)	18·4 (9·0 - 37·8)	4·1 (1·3 - 12·8)	2·9 (1·4 - 5·7)	2·4 (1·1 - 5·2)
**<20y**	3·2 (1·7 - 5·9)	4·4 (1·8 - 10·6)	1·8 (0·6 - 5·8)	-	1·6 (0·6 - 4·4)	20·4 (3·9 - 107·3)	-	1·3 (0·2 - 9·6)	1·5 (0·2 - 11·2)

Risk ratios for the association between history of bacterial meningitis and NDI/education outcomes were estimated using a modified Poisson regression model. Abbreviation: RR=risk ratio.

Figure S1. Health care use among children with bacterial meningitis (red bars) and among children in the comparison cohort (blue bars).

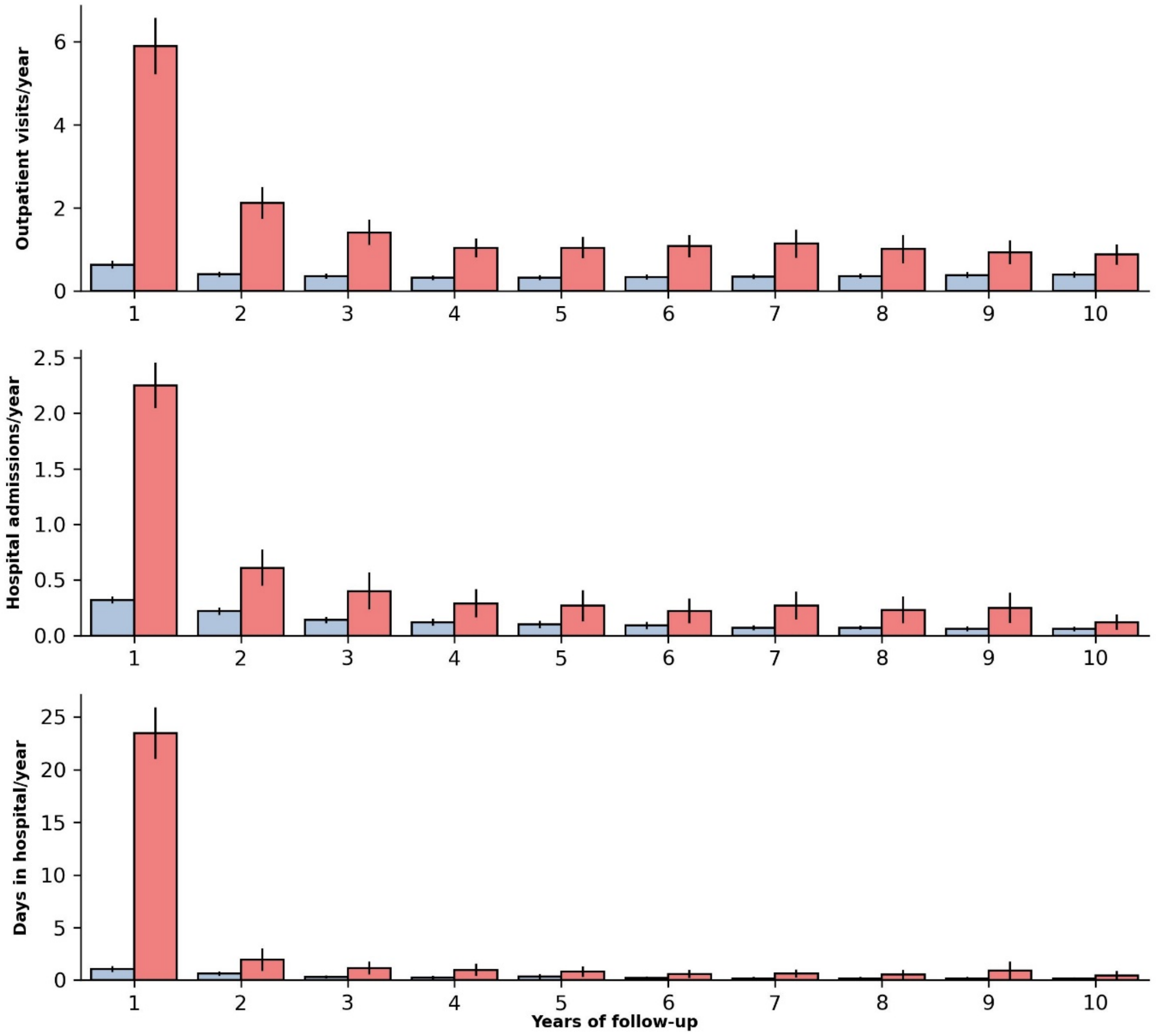

Figure S2. Household income for families of children with bacterial meningitis (red bars) and for families of children without bacterial meningitis (blue bars).

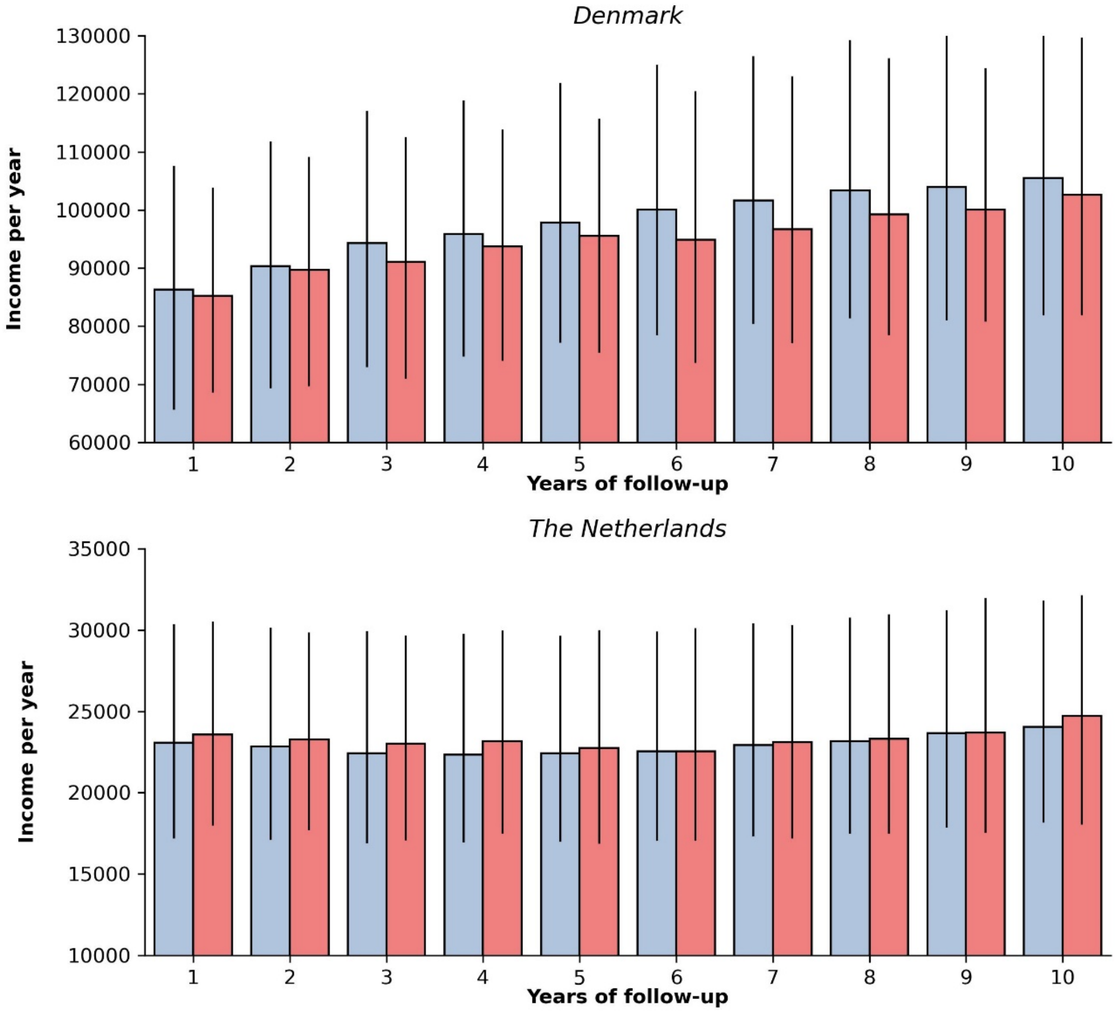

Household income in the Netherlands was adjusted for family size and taxes, so incomes should not be directly compared between the two countries.

Figure S3. Household income. In the top two rows, household income for families of children with bacterial meningitis (red bars) and for families of comparison cohort members (blue bars), stratified by NDI diagnosis or death at any age for the bacterial meningitis cohort, are presented. The bottom row shows comparisons of household income only including families of children with a history of bacterial meningitis: red bars correspond to families of children who died or developed NDI; pink bars correspond to families of children who developed bacterial meningitis but did not die or develop NDI.

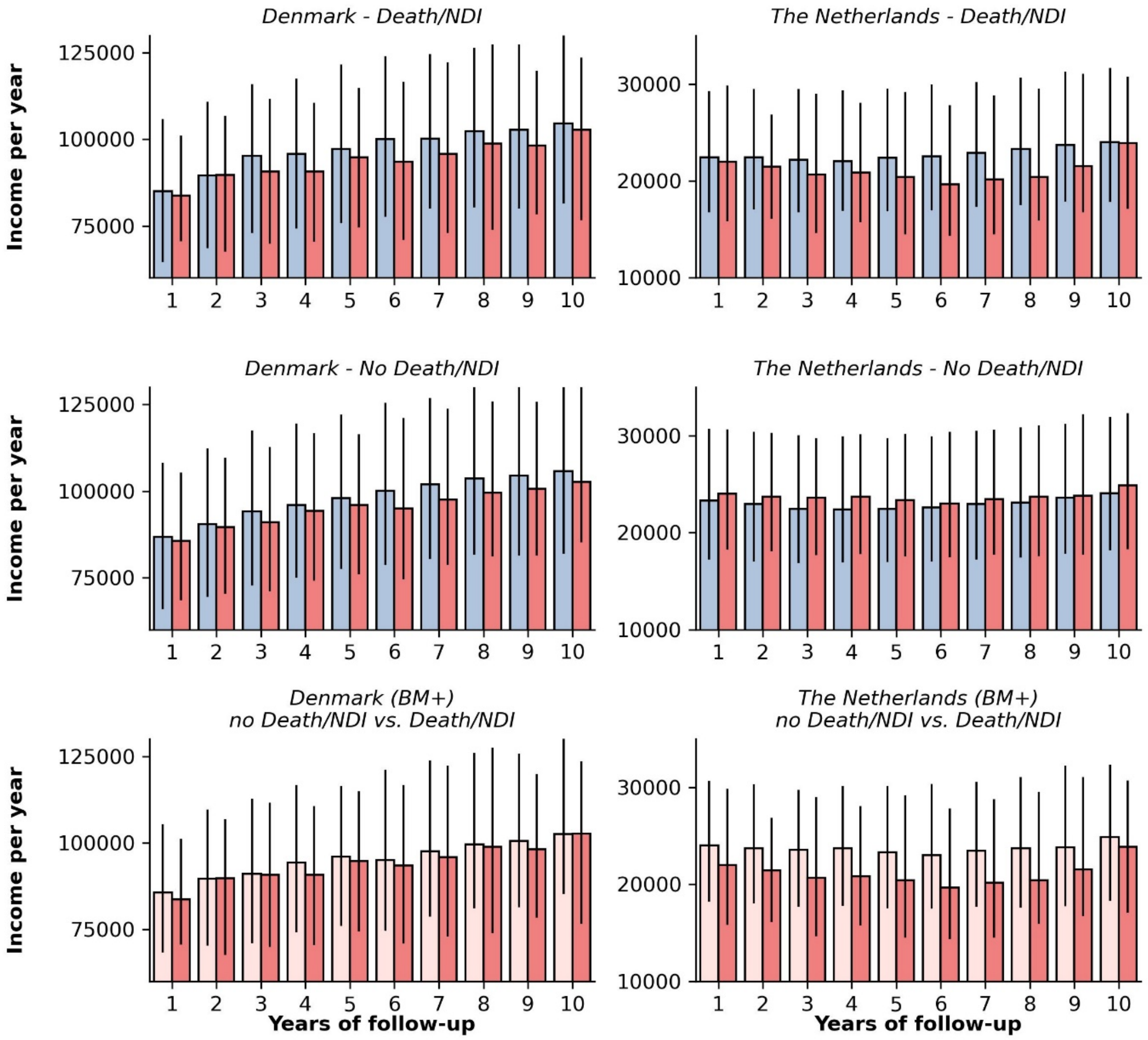

Household income in the Netherlands was adjusted for family size and taxes, so incomes should not be directly compared between the two countries.

References

1. Schmidt M, Pedersen L, Sorensen HT. The Danish Civil Registration System as a tool in epidemiology. Eur J Epidemiol. 2014;29(8):541-9.
2. Schmidt M, Schmidt SA, Sandegaard JL, Ehrenstein V, Pedersen L, Sorensen HT. The Danish National Patient Registry: a review of content, data quality, and research potential. Clin Epidemiol. 2015;7:449-90.
3. Mors O, Perto GP, Mortensen PB. The Danish Psychiatric Central Research Register. Scand J Public Health. 2011;39(7 Suppl):54-7.
4. Baadsgaard M, Quitzau J. Danish registers on personal income and transfer payments. Scand J Public Health. 2011;39(7 Suppl):103-5.
5. European Central Bank Statistical Data Warehouse 2020. (accessed July 14, 2020).
6. Netherlands Reference Laboratory for Bacterial Meningitis, Amsterdam UMC/RIVM, University of Amsterdam, Department of Medical Microbiology and Infection Prevention, Amsterdam Infection and Immunity, Amsterdam, Netherlands.
7. Statistics Netherlands. StatLine, The Hague/Heerlen, 2020 (Assessed December 17, 2020, at http://www.cbs.nl).
8. PeriNed. Perinatale Zorg in Nederland (2000-2019) annual reports (2000–2019). Utrecht, 2020. https://www.perined.nl/onderwerpen/publicaties-perined/jaarboek-zorg.
9. Eskes M, Ensing S, Groenendaal F, Abu-Hanna A, Ravelli A. The risk of intrapartum/neonatal mortality and morbidity following birth at 37 weeks of gestation: a nationwide cohort study. BJOG. 2019;126(10):1252-7.
